# Histoire des Marais, et des Maladies causées par les Emanations des Eaux Stagnantes

**Published:** 1825-10-01

**Authors:** 


### MEDICO-CHIRURGICAL

### REVIEW.

Vol. VII.] **Analytical Series.** [No. 22.

" Nec tibi quid liceat sed quid fecisse decebit

" Occurrat, mentemque domat respectus honesti."

Vol. III.] OCTOBER 1, 1825. [No. 6.

[NEW SERIES.]

### I.

*Histoire des Marais, et des Maladies causées par les Emanations des Eaux Stagnantes. Ouvrage qui a obtenu le Prix mis au Concours par la Société Royale des Sciences, &c. d'Orleans.* Par T. B. Monfalcon, Docteur en Médécine, &c. 1 vol. 8vo. pp. 503. Paris, 1824.

THE surface of our planet presents so many blots and beauties, that it would be easy for an ardent imagination to paint it, and with some fidelity too, as a frightful chaos or a terrestrial paradise, according to the tenor of his mind at the time. Man himself is modified by the soil on which he lives—by the dust of which he is composed! The well-formed Englishman, with

" Pride in his port, defiance in his eye,"

offers a striking contrast with the Cretin of the Valais, or the wretched charbonier of the Pontine fens. Yet these frightful inequalities must be attributed almost entirely to the influence of climate and soil. And not these physical effects on his organization alone, but a whole train of the most devastating maladies to which man is subject, must be traced to emanations from the ground which he cultivates, or the lakes, swamps, or marshes, in his vicinity. Of all the elements, water is that which appears to be the most efficient agent in the production of morbific causes. In all countries, though not equally so, it exercises a hostile influence on the health of man, whether falling in rains, stagnating in marshes, or exhaling in vapours from the surface of the earth—at least so it is thought.

In the state of exhalation, however, it is very improbable that water is any other way concerned than merely as *conveying* the deleterious agent, whatever that may be, into the air—and, in its state of stagnation, it is probably only operative, as the grand agent of decomposition, and as facilitating the extrication of deleterious emanations. However this may be, the history of marshes and of their noxious influence on human health, is a most interesting portion of medical topography, and well deserving of a separate volume for its development. M. Monfalcon, in the performance under review, has evinced very considerable research in the collection of facts, and much judgment in the arrangement of them.

In his introduction he draws an affecting, but not exaggerated, picture of the wretched condition to which the inhabitants of marshy and unhealthy countries are doomed. Disinherited, as it were, by Nature, these people drink only of life's bitterest cup. The fatal influence of the air in which they vegetate, and of their moral and physical habitudes is strongly pourtrayed in their features—modifying, in an extraordinary degree, their functions and their faculties. They are born valetudinarians, and become decrepid by the time that the inhabitants of healthy countries have arrived at the age of vigour. Youth for them has no enjoyment—age nothing but sufferings! It is hardly necessary to say that their intellectual faculties are degenerate in proportion to their physical organization.

Our author enters into the question, whether the atmospheric constitution of marshy countries is now the same as it was formerly—whether or not the climates, the seasons, the temperature, have changed. In respect to this last, (temperature) the most opposite opinions have been maintained—and each supported by apparently strong facts. That the temperature is *increased* is supposed to be proved by the writings of some poets and historians among the ancients. The Euxine Sea does not now freeze, as it is said by Ovid to have done, two thousand years ago. Juvenal talks of the frozen Tyber, &c. On the other hand, there are not wanting facts to support the opposite hypothesis. The olive has deserted France, where it used to grow—and emigrated farther south, to a warmer soil. The crusts of ice and snow on the Swiss mountains have increased rather than decreased in thickness. But, contrary to both these conflicting hypotheses, the great mass of descriptions recorded by our earliest poets and historians, are found to be faithful portraits to the present hour. Snow falls in Ithaca, as it did in the time of Homer. The temperature of

Palestine is the same as it was in the days of Job—and those who travel through Thrace, Greece, and Germany, verify the observations made on those climates by Tacitus, Plutarch, and Xenophon.

For our own parts, we believe that no material change of climate, temperature, or seasons, has taken place, *generally*, on the face of our planet, since those great revolutions that raised mountains from the bottom of the ocean, or heaved the sea over our highest hills—but we think there can be no question respecting the fact of very considerable local changes of climate in certain countries or parts of countries. In the memory of those now living, a considerable amelioration has taken place in the rigour of our own winters. There is not near such an average of frost and snow now in England as there was fifty or sixty years ago.

Previously to entering on the work, our author begs to be understood as applying the term *miasm or miasma* to those morbific matters that emanate from the *living bodies* of men or animals, in a state of *disease*, and the pathological action of which on the human frame he denominates *contagion*. The term *putrid emanations*, he applies to the matters issuing from privies, cemeteries, shambles, certain manufactories, &c. &c. and *marsh effluvium* to those morbific agents that rise from swamps, stagnant waters, and soils—the pathological action of which is denominated *infection*. To these definitions and distinctions we have no objection. Indeed, it would be well for science if authors were more precise in the terms which they use, and more explicit in the sense which they attach to them.

In the first chapter, our author gives a description of the formation of lakes and morasses, but it is unnecessary to say any thing on that subject here. When once formed, they are presented to the medical eye under two principal characters—1*mo.* as consisting of dirty stagnant water, from whence a great evaporation of humidity takes place into the air, especially in hot weather—2*ndo.* as containing a considerable quantity of vegeto-animal substances in a state of decomposition, giving rise to emanations of a specific nature.

These emanations, even granting that they are essentially the same in all places, produce very different effects, according to climate, constitution, modes of life, and many other circumstances. They are much more formidable in a hot, than in a temperate or cold country. They will cause a tertian fever in Germany—a petechial in Hungary—the yellow fever in Antigua—the jungle fever in Ceylon—the fatal Batavian fever in the Island of Java, &c. lastly, according to our author, they will produce the true plague in Egypt and Turkey. The vicinity of marshes in the torrid zone is often sufficient to render vast tracts of country uninhabitable. They have almost annihilated the population of St. Lucie—an isle which the Caribs themselves have deserted. The yellow fever is well known to take up its abode in northern countries during a temperature approaching the tropical scale. It is naturalised at New York, Philadelphia, and other sea-ports of America—almost exclusively prevailing in those parts of the said cities which are low and dirty—especially along the banks of the rivers, where quays, &c. are formed.

The waters of marshes have been examined with great care by physicians and chemists, but without much success. All that is certain is, that they contain vegeto-animal remains in a state of decomposition—and that when portions of their bottoms become exposed to the rays of a powerful sun, a morbific principle is exhaled that causes fevers, dysentery, and various derangements of the internal organs. Passing over the idle conjectures and theories that have been formed respecting the nature of marsh emanations, we come to the question, *are they all of the same nature?* M. Deveze, among others, long anticipated the new doctrine, as it is called, of some teachers and writers in this country, that the same miasm or exhalation from the bodies of men or the surface of the earth, produced indifferently plague, typhus, ague, dysentery, and many other forms of fever. To this sweeping doctrine we would object that, as we know nothing of the real nature of the marsh emanations, we can only argue or judge by their physiological or pathological *effects* on the animal economy. These, we maintain, are different in different localities. The fever of Batavia differs from the fever of Walcheren—the fever of Antigua from the fevers of the Ganges—and all these differ materially from the plague of the Levant. These are facts which cannot be denied, but by theorists who bend facts to support hypotheses. Nor is it likely that the emanations from putrefying animal and vegetable substances in a *marsh*, should be the same as those which issue from *living bodies* in a state of disease. M. Monfalcon cannot bring himself to the opinion, that marsh and human effluvia are of the same nature, though he hesitates in making any difference between the marsh emanations of different countries and localities. How he can reconcile this identity of cause in marsh fevers with dissimilarity of effects, he does not well explain. The dissimilarity of effects is pretty unequivocally admitted in the following passage: "Les fièvres intermittentes de Walcheren ne ressemblent presqu'en rien à celles de Bresken, situé sur l'autre rive de l'Escaut, au temoignage de M. Ferrus. Les fièvres des Marais Pontins diffèrent de celles qu'on voit aux Antilles, et celles des Marais de Rochefort de celles de la Brenne."

Our author dedicates a chapter to the exhalation, ascension, fall, condensation, and transportation of marsh exhalations, taken principally from works on tropical climates, published in this country. We shall therefore pass this chapter over unnoticed.

The succeeding chapter is on the channel by which febrific emanations are received. He thinks, and with reason, that it is not through any exclusive channel, as the lungs, the stomach, the skin—but that it is through the medium of all absorbing surfaces, internal and external. He also admits, with Dr. Johnson, that, under particular circumstances, these emanations appear to act directly on the brain and nervous system.

In respect to the period of incubation, that is, the interval between exposure to marsh poison, and its ostensible effects on the system, we only know that the greatest variety prevails—from a few hours to many months—even a year or more. M. Monfalcon has collected several authentic facts, illustrative of this point, chiefly from English writers, but none throwing any new light on the subject.

We now come to the second part of the work under review, containing a dissertation on the physiological and pathological action of marsh emanations on man and animals.

Man, observes Monfalcon, in marshy countries, is of small stature, and often deformed from infancy, either in the trunk or members, with a remarkable disproportion in the splanchnic cavities. His skin is thin, pallid, and often covered with spots of an earthy appearance. His muscles are flabby, his fat of a watery nature—his hair and beard fair—his complexion pale and sickly-looking—his physiognomy exhibiting, even in youth, indications of age, as well as of melancholy, apathy, and hebetude. His abdomen is prominent, in consequence of the disproportioned development of the abdominal viscera. At twenty-five, the work of decay commences—and the great majority die between the age of 35 and 50 years! It is at this epoch that the endemic fevers rage with redoubled force—that chronic phlegmasiæ of the viscera, obstructions, and dropsies, become established and make rapid progress. A few—a very few, have the good fortune to pass this critical period, and then attain a tolerable and healthy old age. They form but exceptions to, and proofs of, the general rule.

Travellers who have visited and observed the inhabitants of the Pontine marshes in Italy, have drawn a most melancholy picture of these wretched people. They have compared them to spectres, and could not find words to convey the impressions which their appearance made on the minds of strangers. M. Pioney assures us that, in the year 1777, when he visited the centre of the Pontine fens, the greater part of the inhabitants presented such an œdematous state of body, that the impression of the fingers would remain on any part for hours together. Many peasants were seen on the roads, apparently asleep. They had, as it were, ceased to exist. He asked one of the unfortunate sojourners in the Campagna di Roma, how he could live in such an unhealthy place? "We do not *live*," he replied, "we *die* here." Such is the wonderful, and almost insuperable attachment to natal soil, however barren or however insalubrious!

Our author has dedicated a chapter to the "duration of life —mortality—marriages—and depopulation" of the inhabitants of marshy countries. The medium term of human existence in such places, has been calculated by Saussure and Price at 26 or 27—but it must vary so much in different localities and periods of time as to defy all calculation. Marriages, however, are as frequent as in the most fertile countries in the world. It is accounted for by the certainty with which the necessaries of life may be obtained at a low price—and the services of the children, which, in such situations, are the true riches of the parents.

Our author closes this chapter with the following passage: "I have thus shewn the physiological action of marsh emanations on the animal economy. It is seen that these emanations permit the existence of a certain degree of health. But if, under such circumstances, an organ happens to be over-excited —if a sudden elevation of temperature happens to increase the activity of the exhalations, then serious disease is called into action in the individual—or, if it be a time of war, the local endemic is exasperated into a destructive epidemic."

*Pathological Action of Marsh Emanations.* It is evident that this must be a continuation of the preceding subject. The term *physiological*, indeed, is hardly proper on this occasion. All the action or actions of marsh exhalations on the human frame must be of a pathological nature. The *former* term, therefore, is only meant to signify the usual or constant deterioration of constitution produced by residence in marshy countries—the *latter* comprehends the prominent or specific diseases of various organs, destroying the life of the individual, or sweeping off a great portion of the population at the periods of epidemic visitations, in the shape of fever, dysentery, and other fatal maladies. In short, as our author briefly says—"l'action pathologique des modificateurs de l'organisme est, dans les pays marécageux, l'exagération de leur action physiologique." M. Monfalcon first adverts to the effects of marshy countries on animals, enzootic and epizootic. He justly observes (and it may lower the pride of man) that there is no special physiology or pathology for the lords of the creation. Veterinary medicine in the stable or cow-house, acknowledges the same laws as the practice of physic in the gilded palace! Sheep, in marshy countries, very often become dropsical—cows are subject to inflammations in the lungs—horses to chronic anginæ, and, it is said, (but on no great authority, the late Mr. Royston) to regular agues. The thing is not impossible; but it must be rare, otherwise so striking a phenomenon would be readily observed and speedily recorded.

In respect to the pathological action of marsh emanations, it has formed the theme of the most ancient, as well as of the most modern writers. Monfalcon appears to be no great admirer of the ancient fathers of physic. "Shall I be permitted," says he, "to point out, in their pretended portraits of diseases, the extreme confusion of symptoms—the absurd methods of treatment—the inanity of their doctrines—and that radical vice of their cases, the want of *post-mortem* examinations." The great abundance of materials, he adds, only forms an obstacle, instead of a facility, to his own labours. Our author goes on to trace a rapid sketch of those numerous epidemics and endemics described and recorded by various authors, for some hundred years back, and which were attributed, and with reason, to febrific exhalations from the soil, whether in a swampy or dry state. In this historical picture, he has drawn pretty freely on writers of this country, and has not passed unnoticed the fatal fever of Walcheren, which consigned so many of our warriors to an untimely grave—or, what was worse, to years of irremediable suffering, ending, at last, in fatal disorganizations. On the history and treatment of that memorable fever, as recorded by Sir Gilbert Blane, (who was at Walcheren but two or three weeks, and knew very little of the disease) M. Monfalcon has passed some severe strictures. He is totally unacquainted, however, with the best accounts published in this country of that fatal endemic, among which we may mention the work of Dr. Dawson, the ingenious author of a late volume on the practice of physic. Although we are very far from defending our Walcheren practitioners from any share of blame on that occasion, yet we cannot but smile at the sapient criticisms of this disciple of Broussais, who can see nothing in the history of the Walcheren fever but proofs of *gastro-enteritis*, which might have been cured by a few leeches and "eau sucrée." God help thee, Monfalcon! Among many other endemics we saw that of Walcheren, from the beginning to the end. Much error undoubtedly prevailed respecting its nature and treatment; but we are quite satisfied that it was one of those overwhelming visitations which defy, in numerous cases, the power of medicine, how judiciously soever it may be administered. It was a sickly autumn (1809) even among the inhabitants of Walcheren and Beveland—and the circumstances in which the British Army was placed, moral and physical, gave a tremendous additional force to the noxious emanations of that vile country. Had M. Broussais been there, with five hundred thousand leeches in his cortêge, he would have made but a very indifferent stand against the Walcheren fever. We shall treat our readers to a passage from Monfalcon, in his own words:—

"Ce serait avec répugnance, avec une peine extrême, que j'indiquerais le traitement absurde qui fut opposé à cette terrible épidémie, s'il ne prouvait combien la théorie influe sur la pratique, et de quelle importance sont les noms donnés aux maladies. Je suppose que Gilbert Blane et Hamilton n'eussent pas appelé l'épidémie de Walcheren fiévre ou typhus, qu'ils eussent vu en elle une phlegmasie, une irritation, ils auraient entièrement changés de méthodes thérapeutiques, au grand avantage des malheureux Anglais. Mais la maladie est appelée du nom de fiévre, et, au mépris des symptômes inflammatoires les plus violens, tout ce que la thérapeutique possède de plus incendiaire est prodigué avec une opiniâtreté déplorable, à des organes excessivement enflammés. Que la maladie de Walcheren n'ait pas été exclusivement une gastro-entérite, je l'accorde, peu importe cette question ici. Mais ce qu'on ne peut révoquer en doute, c'est la phlegmasie fortement prononcée d'un grand nombre d'organes; l'observation clinique la démontrait; Hamilton la vit exprimée en caractères terribles dans les cadavres, et il continua d'appeler du nom de fiévre la maladie de Walcheren. Elle était, certes, la plus violente des inflammations, voyons comme elle fut traité.

"Lorsque l'irritation des viscéres était à son plus haut dégré d'intensité, Gilbert Blane donnait l'inévitable calomélas, des sels neutres, le quinquina. Si le malheureux estomac rejetait l'ecorce du Pérou, on prescrivait des substances améres, aromatique, et l'opium, la rhubarbe, l'oxide de zinc, surtout l'arsenic. Hamilton commençait généralement par un cathartique, puis venaient les diverses préparations de quinquina aux plus hautes doses possibles. Si ce stimulant manquait son effet, ce qui lui arrivait souvent, on l'associait au sous-muriate de mercure, et aux cathartiques. Le sulfate de cuivre et celui de zinc ne furent pas épargnés (propres expressions d'Hamilton ;) il en fut de même de l'antimoine, de la confection aromatique de la pharmacopée de Londres, du camphre, de l'ammoniaque, de l'acide nitrique, de la gomme gutte. Il ne manque, à cette liste de poisons, que les acides prussique et fluoborique." 176.

The jet of Monfalcon's researches and observations, in this chapter, is to prove that the great pathological feature of marsh fevers is gastritis or enteritis—" maladies dont la *partie fondamentale* était une gastrite ou enterite." Now, although we grant that inflammations of the stomach, intestines, or other viscera, are generally found after death, yet we cannot but admire the curious way in which they are usually removed in the successful cases, namely, by bark and tonics. In the common form of intermittent, they may be very certainly cured in this way, without any evacuation. In the more severe form of remittent, they will frequently yield to the same treatment, guarding important viscera, in the mean time, by suitable means. And yet the Broussaians will still maintain that those are bonâ fide *inflammations* all the while! The following are the proofs which our author offers of the " *doctrine physiologique.*"

" *Preludes of the Disease.* General malaise—restlessness—anxiety—cephalalgia—cold chills, followed by heat.

" *Signs of Gastro-Intestinal Irritation.* Loss of appetite—ardent thirst—distaste for animal food—bitter taste in the mouth—dry tongue, of various colours, and coated in various ways—aphthæ in the fauces—pain in the stomach—heat in the epigastrium—nausea—vomiting of watery, glairy, bilious, or black matters—diarrhœa, dysentery, or cholera morbus—expulsion of worms from the mouth and anus—obstructions of the liver or spleen—icterus—dropsy.

" *Sympathetic Irritations.* Cephalalgia—pulsating pain in the cranium—various disturbances of the intellectual functions—delirium—symptoms of arachnitis or encephalitis, as spasmodic movements, convulsions, præcordial anxiety—smallness, inequality, or intermissions of the pulse—petechiæ, or other affections of the skin.

" *Post-mortem Appearances.* Livid, red, or black spots on the mucous membrane of the stomach and bowels—ulceration, inflammation, or even gangrene of this membrane—tumefaction, inflammation, or disorganization of the liver or spleen—lesions of the peritoneal sheet—inflammation and its consequences in some or all of the other great organs of the body, in the head, chest, and abdomen.

" *Treatment.* This is in harmony with the humoral and entological doctrines. The stomach and intestines are a prey to the most ardent inflammation, *which inflammation becomes periodically exasperated, shewing remissions or intermissions.* But men will not call this an inflammation—they call it a fever; and for curing this fever, they exhibit emetics, purgatives, bark, wine, and stimulants."

In the 14th and 15th chapters of the second part of M. Monfalcon's work, the doctrine of fever is discussed in a way which is calculated to give the English reader a clearer idea of the new physiological doctrine than any thing that has yet appeared in an English dress. On this account, we shall present them with a condensed translation of these chapters, and trust they will not consider it lost time in perusing and reflecting on this important topic.

The 15th chapter (p. 294) commences with the question—" *are fevers general or local diseases?*" He first states the arguments brought forward by those who maintain the existence of idiopathic fever; then the physiological doctrine—and, lastly, gives his own judgment in the case.

1. The Entologists* say that fever is a general affection of the whole system. Look at the symptoms. You see *several* functions disordered from the very commencement—and *all* ultimately deranged. In short, one of the characters of fever is to have its seat constantly multiplied. In one case we see the brain, in another the lungs, in a third some of the abdominal viscera bearing the principal onus of the disease. The febrile movement not only precedes, but often survives any local affection.

But in dissection we find, in a certain number of bodies, the mucous membrane of the stomach or bowels presenting redness, thickenings, or even ulcerations. Hence the exclusive doctrine of the school of Broussais, that the phenomena of fe-

---

* We think the supporters of the new doctrine, that fever is an inflammation of a particular part—in short, that it is a *thing* on which we may put our finger, as it were, are more entitled to the denomination of *entologists* than those who maintain fever to be an assemblage of symptoms resulting from a general affection of all the organs and their functions.—*Ed.*

ver are the result of local irritation. But, say the Entologists, this theory is untenable. In many cases where patients have died of fever, the mucous membrane of the stomach and bowels is found perfectly natural. In many other instances, only a slight redness, of a very equivocal nature, is perceived—and finally only a small number, comparatively, present unquestionable traces of inflammation—an inflammation which we have more reason to think a consequence, than a cause of the fever. How then are we to believe in the existence of inflammations which leave no trace after death? Do we find this the case in other and common inflammations? No, certes. Again, the redness which we often see on the surface of the mucous membrane, after death, may be produced by the *stasis* of the blood in the vessels, *in articulo mortis*—in short, it may be a purely cadaveric phenomenon. Such appearances have been seen in men who have been killed by falls when in perfect health—in criminals after execution—in dogs who had been submitted to physiological experiments. Was it to be supposed that these men and animals had gastro-enteritis? Further, if we admit these rednesses on the mucous membranes to be the cause of the febrile phenomena, what a tremendous disproportion do we observe between the cause and the effect. Many have died with most violent symptoms, where only a slight redness could be seen on the mucous membrane, while others have died with symptoms of extremely feeble irritation, and, on dissection, have presented enormous ulcerations in the stomach and bowels. Where then is the proportion between the supposed cause and effect?

But it may be asked—"how come these red patches, inflammations, or ulcerations, in the mucous membrane of the bowels?" To this the Entologists answer that, in fever, the appetite is lost, and the function of digestion impaired, or altogether destroyed. Whatever matters, therefore are lurking in the primæ viæ, would undergo the chemical decompositions that take place out of the body, and thus many sources of irritation, or even inflammation, would be formed. Add to this that, in fever, all the secretions, salivary, gastric, intestinal, biliary, perspiratory, and even the urinary, are depraved, and, consequently, the mucous membrane of the stomach and bowels is liable to be acted on by the acrimony of its own disordered secretions.

In the new, or physiological system, the cause of adynamic fever is always placed in the gastro-intestinal lining:—and yet, in a great number of these cases, there is no pain in the abdomen or epigastrium, even when considerable pressure is made on those parts. Can the stomach or intestines be inflamed without tenderness on pressure?

Look at the treatment of these pretended gastro-enterites. Although the practice of stimulation may have been carried (and no doubt it was so) to an extravagant length, yet every man must have seen numerous cases of typhus and other adynamic fevers treated, from first to last, with wine, bark, and other stimulants, and yet with very frequent recovery. Is it possible that any case of inflammation of the stomach and bowels could have recovered under such treatment?

But granting, for the sake of argument, say the Entologists, that putrid and adynamic fevers are sometimes, nay, often, dependent on inflammation of the gastro-intestinal lining membrane, what can be said of *intermittents*, in which there is a clear apyrectic period every second day. If these also be cases of gastro-enteritis, ought they to be cured by the administration of sulphate of quinine or arsenical solution, taken not only in the apyrectic interval, but during the very height of the paroxysm? What kind of inflammation must that be which explodes, as it were, the moment the clock strikes a particular hour, and vanishes the moment the clock strikes another hour —and this for days or weeks together? What kind of inflammation (we would ask) is that which, every second day, terminates in a *profuse perspiration* from head to foot, and yet is renewed, after an interval of 48 hours, with the symptoms as before, and so on? Do we see real and unequivocal *inflammations* pursue this course? Never. Are the causes of these *intermittent phlegmasiæ* (if such an expression be not a solecism in medical language) of a periodical or intermittent nature? No. They do not accomodate themselves to any particular theory.*

---

* One or two of the arguments on the above side of the question have been furnished by ourselves; for although we allow Monfalcon to be, upon the whole, a fair propounder of the arguments on both sides, it will soon be seen that he is the advocate of the Broussaian school. It is, therefore, incumbent on us to counteract this bias of our author, by fully developing the cause of the Entologists, and detecting the fallacies or sophistries of the new school.

We shall add one more objection to the gastro-enteritic doctrine, before we close the Entologic arguments. The school of Broussais say, that fevers are *caused* by irritation and inflammation of the mucous membrane of the stomach and bowels. Now, we appeal to the clinical experience of our brethren, whether, in the great majority of fever cases—typhus, for example, the bowels are not more inclined to be torpid, and with difficulty acted on by purgatives, than irritable or relaxed. We ask, would this be the case if gastro-enteritis were invariably the pathological condition of the parts? —*Rev.*

2. The Doctrine of Broussais and his disciples is propounded and enforced with all the zeal, energy, and talents, which that celebrated school can bring into the field.

We can, say they, present the most convincing proof of the truth of our doctrines, in the constantly increasing number of disciples, and of converts to our cause. Pinel, the Entological chieftain, now says, that he attaches to each order of his fevers a local irritation as the cause. Some of his disciples claim for Pinel the honour of being the original discoverer of the new Physiological doctrine. "In this respect, (says the modest and unassuming Monfalcon, as advocate of Broussais) our cause is completely triumphant. The fevers considered idiopathic, or general affections of the whole system, before the time of Broussais, no longer appear so *to any but vulgar practitioners, blindly attached to their superannuated ideas, by routine, senility, or the habit of irreflexion.*" 303.*

But to facts. "If (say the new school) fevers were general affections of the whole system, how is it that they leave traces of their existence in the abdominal viscera *only*." "If these idiopathic fevers are capable of producing inflammations, how is it that the peritoneum, the pleura, and the lungs escape?"† Again, it is plain that fever produced by gastro-enteritis may be easily mistaken for a low or adynamic fever, since the Entologists themselves cannot distinguish the fever resulting from inflammation of the brain, lungs, or other vital organ, from their pretended idiopathic fevers.‡

*Death, say the Broussaians, is not the result of the local disorder caused by irritation. To calculate the danger of an inflammation by the extent which it occupies, or the organic change which it leaves, is to err against the soundest laws of physiology. A disease is dangerous only in proportion to the*

* Such is the mild and modest, and ingenuous set out of Monfalcon. But, if such be a true state of the case, where is our author's wisdom in arraying in frowning order of battle, the whole of the illuminati of the profession, against a few vulgar, superannuated, and unreflecting practitioners? This is killing a butterfly on a wheel with a vengeance! But Monfalcon by no means holds these despicable antagonists so cheap as he pretends to do.—*Rev.*

† We deny the facts, or rather the fictions, contained in the above passage. The inflammations in fevers are not confined to the abdominal viscera, and especially the mucous membranes of the intestines. We assert that, taking the average of a thousand dissections of typhus fever, there will be found traces of inflammation *as often* in other parts of the system as in the said mucous membranes.—*Rev.*

‡ This again we deny. With the exception of phrenitis, no observant practitioner will confound the symptomatic fever of other inflammations with that of typhus for example.

*nature, number, and intensity of the sympathetic disorders occasioned by the local phlegmasia. The latter may be very circumscribed, and yet its influence on the animal economy may be shewn with frightful energy.* A man dies of putrid fever—his body is opened—some patches of redness are found on the gastro-intestinal mucous membrane! Assuredly the fatal issue is not to be attributed to these patches of redness. But the local irritation has acted violently on the brain, the lungs, the heart—and these sympathetic disorders were the cause of death. These disordered functions of distant organs often destroy life before the local cause of irritation has had time to produce any change in the parts affected that could be appreciable after death.*†

In other cases, the constitution has power to resist the first storm of the sympathetic disorder, and the patient dies at an advanced period of the local affection. In such cases, we find a corresponding advance in the said local affection, amounting, sometimes to extensive ulcerations, Death supervenes at various intermediate epochs between these two extremes, according to the degree of nervous susceptibility in the individual. That death (say the school of Broussais) should take place in adynamic or low fevers, before the gastro-enteritis should have time to form so as to leave traces of its existence, is no more wonderful than the fact which we every day witness of death from pleurisy or peritoneal inflammation, where dissection can detect no trace of inflammation in the said pleural or peritoneal membranes.‡

* No objection can be made to these statements. But, if we were to admit the explanation which follows the above passage, and which we have marked in Italics, then there would be an end to all argument, for proofs by symptoms and by dissections are of no use.

† If we admit such airy doctrines as these, it must be against the celebrated maxim—" de non apparentibus et non existentibus eadam est ratio." And to what does this doctrine amount, after all? The miasm of typhus, or plague, or marsh fever, is received into the stomach, and there produces a local irritation. This local irritation produces a general affection or fever, through the medium of the brain or nervous system, which *general disorder* destroys life even before any inflammation has arisen in the original seat of the miasm. Is it not evident that this general fever, when once kindled up, becomes independent of the original local application of the poison, in the same way that the variolous fever and eruption become quite independent of the original inoculation in the arm, or introduction into the stomach of the variolous poison? In this respect we might subscribe to the doctrine of Broussais, provided he could prove that the mucous membrane of the digestive tube was *always* the first recipient of the febrific miasm or cause.—*Rev.*

‡ Here is another Broussaian fact, of every day occurrence, (as it is said) which we deny. We have never seen a fatal case of pleuritis or peritonitis

The red stripes or spots (they observe) which are seen in the bowels of those who die of idiopathic fever, are the result of inflammation, and not a mere mechanical stasis of the blood. If they have been seen in the bodies of men and animals suddenly destroyed, why should not these men and animals have been labouring under gastro enteritis at the time of their death? "Pourquoi les uns et les autres n'aurent-ils pas eu une gastro-entérite au moment où ils ont perdu la vie?" Men condemned to death are assailed, of course, by the most depressing passions, and frequently have recourse to the intemperate use of drink after condemnation. It is not unreasonable, therefore, to suppose they may be affected with *gastro-enteritis* at the time.*

It is too late in the day for the Entologists to say that the contents of the primæ viæ, and the secretions of the abdominal viscera, become vitiated and changed in fever. There is no such thing. "La fièvre ne saurait changer directement la composition de nos liquids.†"

But, say the Entologists, in a great number of putrid fevers there are no abdominal pains—no tenderness on pressure. Thanks to M. Broussais, who has solved this pathological problem. He has proved that the intestines possess very little sensibility. "Il a demontré que les intestins possédaient fort pue de sensibilité." 311.

---

without *post-mortem* marks of the inflammation. If others have seen such cases let them state them. We believe, indeed, that such a *painful impression* may be made on the *nervous system* of the stomach, or other very sensible part, as to suddenly destroy life, leaving no cognizable change in the part:—but this is a very different thing from inflammation.—*Rev.*

* There is no resisting the ingenuity of such arguments. They might have added that animals destined to be killed for the purpose of physiological experiments, have a wonderful foresight of their fate—undergo the most melancholy moral reflexions—and, no doubt, commit great excesses in strong drink under such circumstances. Hence the *gastro-enterites* that are observed in them on dissection.

† It is really amusing to observe the assertions made by the school of Broussais in the teeth of facts and experience. If there be such a thing as a truth in medical science, it is this vitiation of the secretions during the febrile state. Who has ever examined the excretions of fever-patients and not come to this conclusion? What is the cause of the good effects of purgatives in fevers, but the constant removal of unhealthy secretions, and the excitement to healthy ones. Why again, we would ask, are these phlogoses and ulcerations so much more frequently found in the mucous membrane of the intestines in France than in England, where (England) so much purgation is employed? If these phlogoses were the causes, not the consequences, of the fever, should they not be increased, rather than diminished, by the purgatives administered in this country? Yet the contrary is the case.

Again. The most intrepid of our adversaries, say the Broussaians, dare not compare the success of their practice with the success of ours.* No scourge has been equal to the abuse of stimulating medicines in the treatment of the pyrexiæ—they have destroyed more than the plague itself. Brunoneanism is a destructive monster that cannot be too soon annihilated.

As for intermittent fevers, they are not the rock on which the "doctrine physiologique" must split. These fevers differ from those of the continued form only in the mode of their development. They arise from "gastro-intestinal *irritation*," which makes all the difference. Why should not the mucous membrane of the digestive tube be subject to *intermittent irritations* as well as the serous, fibrous, and cutaneous structures? Nothing is more common than to see intermitting fevers converted into remitting or continued fevers, and *vice versa.* Is it, therefore, necessary to suppose that, in these conversions, the seat of the disease has changed its place?†

Pathological anatomy proves the identity of intermitting and continued fevers. The same post-mortem appearances are found in both, though not so frequently in the former as in the latter. As to the *type* of a fever, it is nothing. "Peu importe le type d'une pyrexie." What is its proximate cause? Why have we those intervals of health between the paroxysms of an ague? This is a problem which we cannot at present

* It has not only been compared—but it has been confidently asserted, from authentic hospital returns, that the success of the new practice is inferior to that of the old. Considering, however, the errors into which the Brunonians were led by their prodigality of stimulation in fevers, we might safely admit the superiority of Broussais's practice, without at all subscribing to the truth of the doctrine on which it is founded. Many a time, in the history of medicine, has a false theory directed a safe practice. But, although we admit (and we do so cheerfully) that the practice of Broussais is infinitely safer than that of Brown, it does not follow that it is the best practice. To treat fever *always* as a local inflammation will be the way to consign many a patient to the grave, who might be saved by a more enlightened and a less exclusive doctrine. We maintain that inflammation does *not always* exist in fever—and, consequently, that eternal leeching is not *always* necessary;—on the other hand, we maintain that, when actual inflammation of an internal organ obtains, (whether as cause or effect) the practice of *leeching* and *eau sucrée* is not *always* to be depended on.

† No. But as you have not proved that the seat of the continued fever is in the mucous membrane of the digestive tube, this reference to continued fever is of no use. Granting that the malaria of Walcheren (for instance) made its first impression on the stomach, and thence produced the phenomena of the fever. That fever became independent of the miasm that first produced it, else, how did it happen that the fever came on at intervals for many years afterwards, when the soldiers were in other and healthy countries?

solve. "Each attack of an ague does not constitute a pathological condition, (etât pathologique) independent of those paroxysms which have preceded and shall follow it. A manner of being, unknown as to its nature, permanent during the apyrexia, connects the paroxysms together, and renews them without the assistance of the action of the irritating cause." "Une maniere d'être inconnue encore dans sa nature, permanente pendant l'apyrexie, les lie, les rappelle sans le concours d'action de la cause irritante."*

In respect to the stumbling block of intermittent fevers being cured by bark and stimulants, though their cause is gastro-intestinal irritation, it is easily got rid of. These tonics are only given during the intermissions when the irritation does not exist, and they, by some means which we are ignorant of, (and which, by the way, it is a vile shame they should ever have exhibited—*Rev.*) prevent the return of the gastro-intestinal irritation.

3. Having now set both sides of the question before his readers, M. Monfalcon unequivocally declares himself "entirely in favour of the new physiological doctrine." At the same time, he does not seem quite easy about some of its parts. He says it is not yet perfect. It has a little exaggerated the attributes of the stomach, at the expence of the brain, and perhaps of some other organs. It has too often, and to too great a degree, merged all organism in affections of the mucous

* This is an important passage, and upon it we shall ground the final overthrow of the new doctrine. By this passage we learn that, although the first paroxysm of an intermittent, which, we will say, takes place on a Monday, is owing to a *gastro-intestinal irritation*; yet that the second paroxysm, on the Wednesday, may take place quite independent of the action of the irritating cause—that is, independent of the *gastro-intestinal irritation*—and that in consequence of "a manner of being, unknown as to its nature." Now we ask the Broussaian school, why may not the first paroxysm occur in consequence of this "manner of being," which obtains during the incubation of the marsh miasm, that is, during the interval between its reception and manifestation in the system, as well as the second paroxysm? What proof do they offer that the Wednesday paroxysm acknowledges a cause different from that of the Monday? And so it is with typhus and many other fevers. Contagion, or other febrific cause, is received into the system—an interval or period of incubation takes place—then the fever is developed in a continued, remittent, or intermittent form, totally independent of the original impression of the poison, on whatever part it first came in contact with. That part, say the stomach, or any other part, as the brain, lungs, liver, &c. may, and frequently does, become inflamed in the course of the fever, but these local affections are no more the cause of the pyrexia, (though they aggravate it) than the inflammation of a small-pox pustule on the arm is the cause of the eruptive fever.

membrane of the stomach and bowels—in short, it is, says our author, like all other human productions, susceptible of improvement. "But still I maintain that there are no idiopathic fevers—and that all the pyrexiæ, continued or intermittent, are, *in their ultimate result*, a local affection—an irritation or an inflammation." "Mais il n'en est pas moins vrais qu'il n'y a pas de fièvres essentielles, et que toutes les pyrexies, continues ou intermittentes, sont, *en dernier resultat*, une maladie locale." The reader will see that there is something equivocal in the phrase *ultimate result;* for there is little doubt that few fevers, comparatively speaking, proceed to a fatal termination, without some local inflammation. It is this local inflammation which constitutes one of the principal sources of danger in all fevers; but this leaves the question still undecided, as to the local affection being the cause of the fever.

M. Monfalcon says that, by adopting this doctrine, we have much to gain and nothing to risk. We do not think so. There are numerous cases of fever which would be decidedly rendered more dangerous by the treatment proper in cases of inflammation of an internal organ. In fact, there is not a stronger proof of the baseless fabric of Broussaiism, than the want of conformity in the treatment of fever and inflammation. We repeat it, that he who treats both alike, in the general run of cases, will either do too much in fever or too little in the phlegmasiæ. At the same time, we are ready to allow that Broussaiism is preferable to Brunonianism. On the continent, especially, it has done much good, because the treatment there adopted for the phlegmasiæ is so very inefficient, compared with the measures employed in this country, that it can seldom do harm in common fever, and may often prevent the supervention of local inflammation. But, in this country, we have seen the practice adopted by the partisans of the phlegmasian doctrine of fever,* do infinite mischief—and render fevers that would have done well under mild treatment, extremely protracted, and sometimes fatal.

4. *Seat of Marsh-Fevers*. Marsh fevers, M. Monfalcon admits, are not always caused by *inflammation*—nor is the inflammation, when it does exist, always located in the mucous surfaces of the alimentary canal. "What physician," says he "of Sologne or Bresse, has not seen fever-patients in these

* In England, fever, it is said, has its domicile in the brain, and the treatment of real phrenitis is no milk and water practice here.—*Rev.*

marshy districts, with the tongue clean, moist, and free from redness—free from thirst, and also from pain in any part of the abdomen, even on pressure?" In other cases, he observes, the affection of the digestive apparatus is very slight or entirely absent, and there is severe irritation or inflammation of the brain, or of some other organ. Our author quotes, with approbation, M. Jourdain, who says that marsh fevers depend on organic lesions of some of the viscera—but seldom on gastro-enteritis*—"that they are prolonged to an indefinite period, if opposed solely by antiphlogistic measures—nay, that they are aggravated by the antiphlogistic treatment." How any man in his senses could suppose that the *cause* of these fevers is inflammation, and yet that they are aggravated by *antiphlogistic* treatment, is beyond our conception! It is, however, not more strange than the argument of Dr. M'Lean, that, because he caught the plague in the Greek pest-house, the plague is necessarily *non-contagious*!!

The remainder of the work under review is on the Hygiène and Therapeutics of marsh-fevers—subjects which are better understood in this country than in France. We shall, therefore, take leave of our author at this point. We have combated such of his opinions as appeared to us erroneous, without detracting from his merits as a learned, an ingenious, and a zealous cultivator of medical science. Our unrestrained intercourse with the French metropolis produces annually a certain return of converts to the school of Broussais; and, as we conceive that exclusive doctrines are calculated to injure the best interests of medical science, we have taken this opportunity of exposing the weakness, not to say the dangerous tendency, of a theory which is epidemic in France, and spreading in this country.

---

### II.

*Observations on Extraction of Diseased Ovaria; illustrated by Plates colored after Nature.* By John Lizars, Surgeon, author of the System of Anatomical Plates, &c. Folio, pp. 24, five coloured plates. Edinburgh, May, 1825.

We have been false prophets—not the first, however, in the world. We expressed our disbelief of the successful removal

* Monfalcon and Jourdain seem to forget—or, at all events, they pass the fact unnoticed, that Ploucquet maintained this identical doctrine more

